# Surgical treatment after hepatic arterial infusion chemotherapy for hepatocellular carcinoma extending into the right atrium

**DOI:** 10.1186/s40792-015-0047-z

**Published:** 2015-06-06

**Authors:** Shintaro Kurahashi, Tsuyoshi Sano, Seiji Natsume, Yoshiki Senda, Hidekazu Yamaura, Yoshitaka Inaba, Yasuhiro Shimizu

**Affiliations:** Department of Gastroenterological Surgery, Aichi Cancer Center Hospital, Kanokoden 1-1, Chikusa-ku, Nagoya, Aichi Japan; Department of Gastroenterological Surgery, Aichi Medical University Hospital, 1-1 Yazakokarimata, Nagakute, Aichi Japan; Department of Interventional Radiology, Aichi Cancer Center Hospital, Kanokoden 1-1, Chikusa-ku, Nagoya, Aichi Japan

**Keywords:** Hepatocellular carcinoma, Tumor thrombus, Inferior vena cava, Hepatectomy, Right atrium

## Abstract

A resected case of hepatocellular carcinoma which extended into the right atrium after treatment with hepatic arterial infusion chemotherapy (HAIC) is described. An 81-year-old man presented with right hypochondralgia. CT demonstrated a hypervascular tumor 11.5 cm in diameter extending into the right atrium through the right hepatic vein. The patient underwent HAIC with 100 mg of cisplatin (CDDP IA-call®) particles three times every month. The tumor showed a marked shrinkage and an involution of the venous thrombus around the orifice of the right hepatic vein. Right hemihepatectomy with tumor thrombectomy was performed as a salvage surgery using a total hepatic vascular exclusion technique. Histologically, the tumor turned into diffuse necrosis and fibrosis, so viable tumor cells were encountered neither in the main tumor nor venous thrombus. The therapeutic effect of HAIC was pathological complete remission. The patient has been doing well for 6 years after the surgery without evidence of tumor recurrence. The salvage operation was safely achievable for the initially unresectable advanced hepatocellular carcinoma extending into the right atrium.

## Background

Hepatectomy for a hepatocellular carcinoma with a hepatic venous tumor thrombus extending into the right atrium (HCC-RA thrombus) is a rare surgical indication for cure. However, surgical resection for HCC-RA thrombus is occasionally performed as an oncological emergency to prevent pulmonary embolism or sudden right heart failure due to tearing away of the fragile tumor thrombus [1–3]. Complete removal of a HCC-RA thrombus usually necessitates a total hepatic vascular exclusion technique [4] or cardiopulmonary bypass [5, 6]. Such an aggressive and invasive surgery must be a heavy burden in patients with impaired liver function, and long-term survival is seldom achievable because of apparent or latent pulmonary metastases in many patients with HCC-RA thrombus.

On the other hand, some chemotherapeutic agents such as cisplatin or sorafenib [7] are potentially effective in the neoadjuvant setting, or multidisciplinary treatments [8] may thereby expand a surgical indication and also prolong survival for patients with HCC-RA thrombus.

We describe an aggressively resected case of HCC-RA thrombus after treatment with hepatic arterial infusion chemotherapy (HAIC) using cisplatin particles, and the patient survives more than 6 years without a tumor recurrence.

## Case presentation

**Fig. 1:**
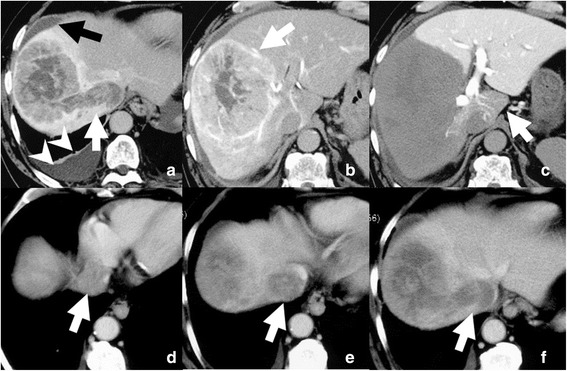
**a** CT during hepatic arteriography shows a heterogenous hypervascular tumor and venous tumor thrombus, corresponding to the right hepatic vein, extending into the IVC (*white arrow*). Ascites (*black arrow*) and pleural effusion (*white arrowheads*) is also documented. **b** CT during hepatic arteriography shows a heterogenous hypervascular tumor measuring 11.5 cm in diameter (*white arrow*). **c** CT during arterial portography shows a tumor defect and hypoperfused caudate lobe (*white arrow*); nevertheless, the caudate branch of the portal vein is patent. This implies congestion of the caudate lobe due to the IVC tumor thrombus. **d**–**f** The tip of the venous tumor thrombus extends into the right atrium (*white arrow*)

An 81-year-old man presented with right subcostal pain. Laboratory examinations showed marked elevation of alpha-fetoprotein (AFP) (195,460 ng/ml) and des-γ-carboxy prothrombin (DCP) (367,000 mAU/ml). The hepatitis C antibody and hepatitis Bs antigen were negative, while the hepatitis Bs antibody was positive. CT demonstrated a hypervascular tumor of 11.5 cm in diameter extending into the RA through the right hepatic vein, with a small amount of pleural effusion and ascites (Fig. 1). Angiography depicted a solitary HCC showing the thread and streak sign corresponding to the venous tumor thrombus from the right hepatic vein to the right atrium (Fig. 1a). CT during arterial portography demonstrated hypoperfusion in the entire caudate lobe, reflecting congestion of the inferior vena cava (IVC) due to the tumor thrombus (Fig. 1c). The diagnosis for this patient was HCC-RA thrombus. Right hemihepatectomy with tumor thrombectomy was considered a highly detrimental procedure for this particular patient at first presentation.

**Fig. 2:**
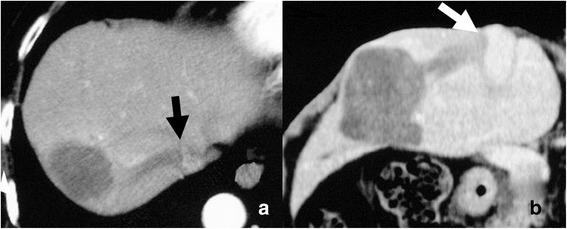
CT after three sessions of hepatic arterial infusion chemotherapy shows marked shrinkage of the main tumor and tumor thrombus. Additionally, the tip of the tumor thrombus (*arrow*) can be confirmed around the orifice of the right hepatic vein. **a** View in the axial plane. **b** View in the coronal plane

The patient underwent HAIC with 100 mg of cisplatin (CDDP IA-call®) particles in 20 min via the right hepatic artery three times every month. Both AFP and DCP decreased to within the normal range after the second HAIC session. CT showed a marked shrinkage of the main tumor and the tumor thrombus. Additionally, the tip of the tumor which had been extended into the right atrium before HAIC could be confirmed around the orifice of the right hepatic vein (Fig. 2). Although both the tumor and thrombus turned into low density with slight marginal enhancement, the viability of the tumor cells could not be definitively assessed on the imaging diagnosis. The maximum diameter of the main tumor changed from 11.5 to 4.8 cm, and a 58.3 % reduction was achieved through the third HAIC session. Accordingly, the therapeutic effect of HAIC was a partial response (PR) to the Response Evaluation Criteria In Solid Tumors (RECIST) criteria [9].

**Fig. 3:**
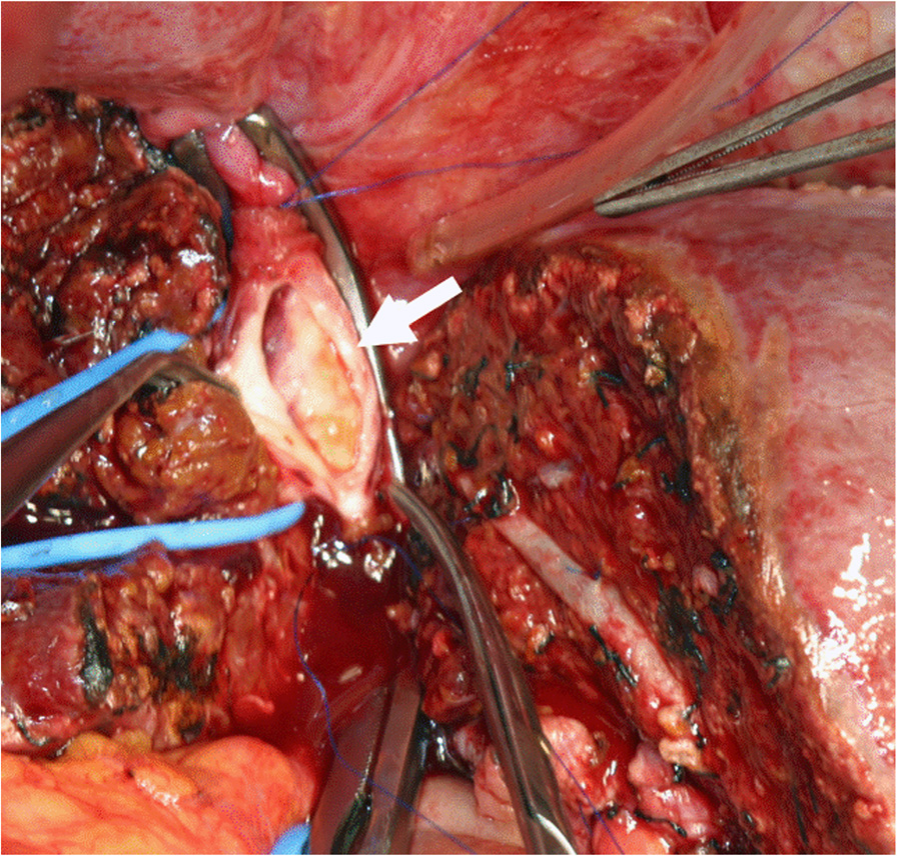
After liver transection through the anterior approach, the right hepatic vein is encircled with tape and the side of the IVC is clamped. After making an incision on the anterior surface of the confluence of the right hepatic vein, the fibrous tumor thrombus (*arrow*) is observed

Estimated liver resection volume excluding the tumor mass after three sessions of HAIC was calculated by CT-volumetry as 27 %, and the ICG retention rate at 15 min improved from 23 to 16 %. We considered that right hemihepatectomy with tumor thrombectomy would be safely achievable in this condition. The liver was transected along the main portal fissure in terms of the anterior approach [10]. Then, the side clamp of the IVC was placed over the base of the right hepatic vein, and the ventral orifice of the right hepatic vein was incised (Fig. 3). The apical part of the fibrous tumor thrombus tightly adhered to the right side wall of the suprahepatic IVC. Right hemihepatectomy with tumor thrombectomy was possible using a total hepatic vascular exclusion (THVE) technique. The operation time was 365 min, and intraoperative blood loss was 3010 ml. The liver inflow occlusion and THVE times were 81 and 21 min, respectively.

**Fig. 4:**
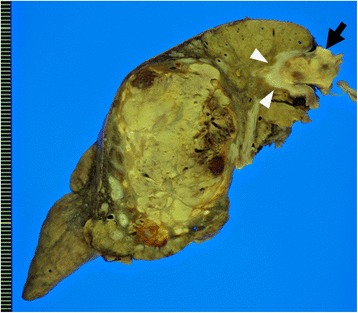
The cut surface of the resected specimen demonstrates a necrotic tumor, 4.5 cm in maximum diameter, and the fibrotic venous thrombus (*arrow*) in the right hepatic vein (*arrowhead*)

**Fig. 5:**
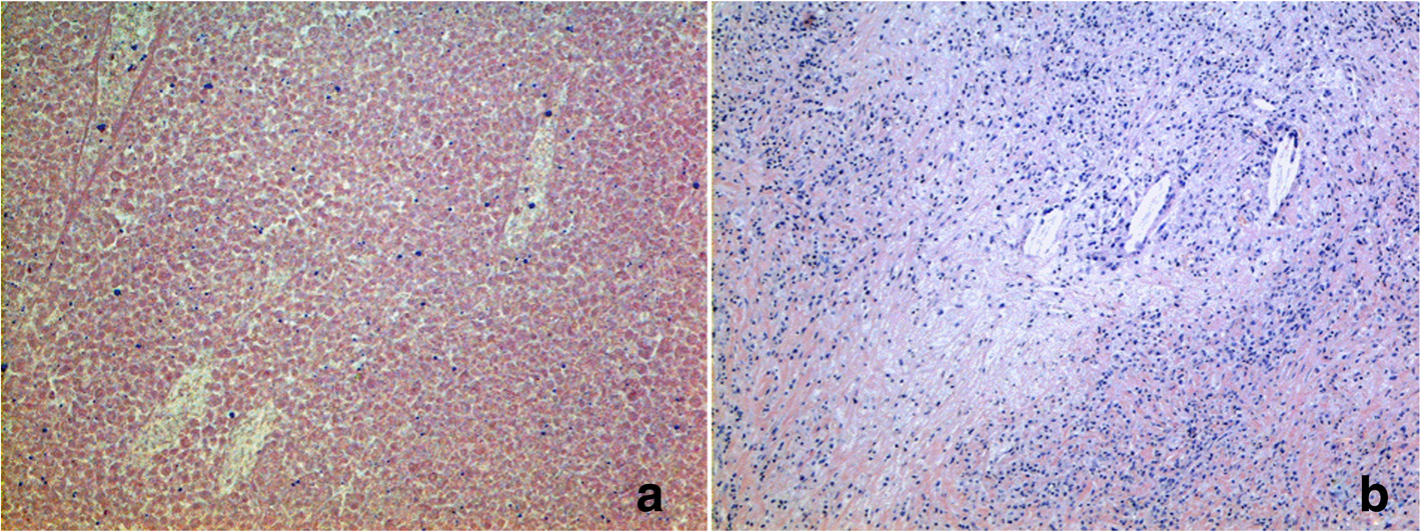
Microscopic findings of the main tumor and venous tumor thrombus are depicted (hematoxylin eosin stain). The main tumor consists of diffuse necrosis and fibrosis (**a**). Viable tumor cells are found neither in the main tumor nor venous tumor thrombus (**a**, **b**)

Macroscopically, the cut surface of the resected specimen showed a necrotic tumor, 4.5 cm in maximum diameter, and the fibrotic venous tumor in the right hepatic vein (Fig. 4). Histologically, the tumors showed diffuse necrosis and fibrosis similar to the macroscopic findings, so viable tumor cells were encountered neither in the main tumor nor venous thrombus in multiple sections (Fig. 5). The therapeutic effect of HAIC was pathological complete remission (CR). The clinical stage was T3bN0M0, stage IIIB, and the pathological stage was pT0N0M0, a stage unknown in terms of the UICC classification (seventh edition) [11], respectively.

Postoperative recovery was uneventful. Without an additional oncologic treatment after surgery until now, the patient has been alive with no recurrence for 6 years.

## Discussion

Compared with portal venous tumor thrombus, hepatic venous tumor thrombus (HVT) extending into the IVC or right atrium is rare; it is reported in 6 % of antemortem HCC with vascular invasions [12]. Surgery for patients with HVT often necessitates complex and advanced surgical skills such as the THVE technique or cardiopulmonary bypass. The burden of the surgical procedure must be especially heavy for HCC patients often associated with impaired liver functional reserve. In addition, prognosis of patients with HCC-RA thrombus is extremely poor even in the case of complete removal of hepatic tumor and thrombus because most of the patients have apparent or latent pulmonary metastases. Although Kaido et al. reported a surgical result of patients with HCC associated with tumor thrombus confined to the inferior vena cava, it did not extend into the right atrium [13]. They reported a 5-year survival rate and median survival time as 30 % and 26 months with both neoadjuvant and adjuvant therapy. Survival after surgical resection for HCC-RA thrombus was reported by only 13 % of a 2-year survival rate with 8.9 months median survival in a literature review [1]. To the best of our knowledge, there have been no case reports of hepatectomy for HCC-RA thrombus with over a 5-year recurrence-free survival. This underscores the exceedingly rare nature of our particular case. We attributed the long-term survival of our case to achieving pathological CR by preoperative HAIC. Spontaneous regression or CR through anti-cancer therapy for HCC can be documented, albeit uncommonly [14–18]. A similar phenomenon must have occurred in this case. Thus, careful observation of the course without surgery may have yielded the same outcome as hepatectomy. However, the precise or definitive assessment of tumor viability as CR is difficult despite recent advances in imaging diagnosis [19, 20]. In this case, our therapeutic evaluation of a tumor using the RECIST criteria was not pathological CR but PR prior to surgery. Assessment of the tumor viability after chemotherapy is not precise and definitive; therefore, we considered that there must be residual viable tumor cells, as cited similar to the following reports: CR of colorectal liver metastases after recent brand new chemotherapy [20] and CR of HCC after transarterial chemoembolization (TACE) [21].

Whether preoperative HAIC is valid for HCC-RA thrombus is controversial, since this strategy has both advantages and disadvantages. A representative failing requiring close attention is pulmonary embolism or sudden right heart failure due to tearing away of the fragile tumor thrombus during preoperative care. On the other hand, preoperative HAIC has some potentially meaningful advantages as follows. First, it may reduce surgical invasiveness owing to the shrinkage of tumor thrombus. In our case, also, the tip of the venous thrombus was markedly decreased around the orifice of the right hepatic vein, so right hemihepatectomy with tumor thrombectomy could be safely achievable as a salvage surgery without cardiopulmonary bypass. Second, the potential benefit of preoperative HAIC includes the opportunity to monitor the response to anti-cancer agents, which is impossible in the adjuvant setting. As several authors showed, hepatectomy alone could not provide long-term survival without effective adjuvant chemotherapy for patients with HCC-RA thrombus [22]. Therefore, it might be possible to avoid unnecessary surgery for patients with little chance of long-term survival by screening the patients with poor response to preoperative HAIC. Moreover, in the current case, we could acquire an unexpected preoperative HAIC effect of improving liver function test possibly due to the tumor shrinkage relieving liver blood congestion. Although the effectiveness of preoperative HAIC for HCC-RA thrombus is controversial, our aggressive strategy will be justified as not palliative but curative by a longer patient survival.

## Conclusions

The most striking point of this case is the achievement of recurrence-free long-term survival over 6 years in patients with HCC-RA thrombus which was considered initially intolerable for a radical surgery.

## Consent

Written informed consent was obtained from the patient for publication of this case report and any accompanying images. A copy of the written consent is available for review by the editor-in-chief of this journal.

## Abbreviation

CT: computed tomography

## References

[1] Sung AD, Chen S, Moslehi J, Scully EP, Prior JM, Loscalzo J (2008). Hepatocellular carcinoma with intracavitary cardiac involvement: a case report and review of the literature. Am J Cardiol..

[2] Nonami T, Nakao A, Harada A, Kaneko T, Kurokawa T, Takagi H (1997). Hepatic resection for hepatocellular carcinoma with a tumor thrombus extending to inferior vena cava. Hepatogastroenterology.

[3] Lin HH, Hsieh CB, Chu HC, Chang WK, Chao YC, Hsieh TY (2007). Acute pulmonary embolism as the first manifestation of hepatocellular carcinoma complicated with tumor thrombi in the inferior vena cava: surgery or not?. Dig Dis Sci..

[4] Bismuth H, Castaing D, Garden J (1989). Major hepatic resection under total vascular exclusion. Ann Surg..

[5] Fujisaki M, Kurihara E, Kikuchi K, Nishikawa K, Uematsu Y (1991). Hepatocellular carcinoma with tumor thrombus extending into the right atrium: report of a successful resection with the use of cardiopulmonary bypass. Surgery.

[6] Wu CC, Hseih S, Ho WM, Tang JS, Liu TJ, P’eng FK (2000). Surgical treatment for recurrent hepatocellular carcinoma with tumor thrombi in right atrium: using cardiopulmonary bypass and deep hypothermic circulatory arrest. J Surg Oncol.

[7] Llovet JM, Ricci S, Mazzaferro V, Hilgard P, Gene E, Blanc JF (2008). SHARP Investigators Study Group. Sorafenib in advanced hepatocellular carcinoma. N Engl J Med.

[8] Chang JY, Ka WS, Chao TY, Liu TW, Chuang TR, Chen LT (2004). Hepatocellular carcinoma with intra-atrial tumor thrombi. A report of three cases responsive to thalidomide treatment and literature review. Oncology.

[9] Therasse P, Arbuck SG, Eisenhauer EA, Wanders J, Kaplan RS, Rubinstein L (2000). New guidelines to evaluate the response to treatment in solid tumors. European Organization for Research and Treatment of Cancer, National Cancer Institute of the United States, National Cancer Institute of Canada. J Natl Cancer Inst.

[10] Liu CL, Fan ST, Cheung ST, Lo CM, Ng IO, Wong J (2006). Anterior approach versus conventional approach right hepatic resection for large hepatocellular carcinoma: a prospective randomized controlled study. Ann Surg.

[11] Sobin LH, Gospodarowicz M, Wittekind C (2009). International Union Against Cancer (UICC): TNM Classification of Malignant Tumors.

[12] Ohwada S, Tanahashi Y, Kawashima Y, Satoh Y, Nakamura S, Kobayashi I (1994). Surgery for tumor thrombi in the right atrium and inferior vena cava of patients with recurrent hepatocellular carcinoma. Hepatogastroenterology.

[13] Kaido T, Ogawa K, Mori A, Fujimoto Y, Ito T, Uemoto S (2013). Usefulness of the Kyoto criteria as expanded selection criteria for liver transplantation for hepatocellular carcinoma. Surgery.

[14] Matsuda M, Shiba S, Asakawa M, Kono H, Fujii H (2009). Complete remission of multiple recurrent hepatocellular carcinomas by oral administration of enteric-coated tegafur/uracil in a patient with huge hepatocellular carcinoma extending to the inferior vena cava after hepatic resection: analysis of mRNA expression of fluoropyrimidine metabolism enzymes in the primary tumor. Int J Clin Oncol.

[15] Nakamura M, Nagano H, Wada H, Noda T, Ota H, Damdinsuren B (2006). A case of hepatocellular carcinoma with multiple lung, spleen, and remnant liver metastasis successfully treated by combination chemotherapy with the novel oral DPD-inhibiting chemotherapeutic drug S-1 and interferon-alpha. J Gastroenterol.

[16] Ishikawa T, Ichida T, Ishimoto Y, Yokoyama J, Nomoto M, Ebe Y (1999). Complete remission of multiple hepatocellular carcinomas associated with hepatitis C virus-related, decompensated liver cirrhosis by oral administration of enteric-coated tegafur/uracil. Am J Gastroenterol.

[17] Wada H, Nagano H, Noda T, Damdinsuren B, Marubashi S, Miyamoto A (2007). Complete remission of hepatocellular carcinoma with portal vein tumor thrombus and lymph node metastases by arterial infusion of 5-fluorouracil and interferon-alpha combination therapy following hepatic resection. J Gastroenterol.

[18] Uenishi T, Hirohashi K, Tanaka H, Ikebe T, Kinoshita H (2000). Spontaneous regression of a large hepatocellular carcinoma with portal vein tumor thrombi: report of a case. Surg Today.

[19] Bargellini I, Vignali C, Cioni R, Petruzzi P, Cicorelli A, Campani D (2010). Hepatocellular carcinoma: CT for tumor response after transarterial chemoembolization in patients exceeding Milan criteria–selection parameter for liver transplantation. Radiology.

[20] Benoist S, Brouquet A, Penna C, Julie′ C, Hajjam ME, Chagnon S (2006). Complete response of colorectal liver metastases after chemotherapy: does it mean cure?. J Clin Oncol.

[21] Takayasu K, Shima Y, Muramatsu Y, Moriyama N, Yamada T, Makuuchi M (1987). Hepatocellular carcinoma: treatment with intraarterial iodized oil with and without chemotherapeutic agents. Radiology.

[22] Lovet JM, Bustamante J, Castells A, Vilana R, Ayuso MC, Sala M (1999). Natural history of untreated nonsurgical hepatocellular carcinoma: rationale for the design and evaluation of therapeutic trials. Hepatology.

